# Editorial: Biofilm-mediated nosocomial infections and its association with antimicrobial resistance: Detection, prevention, and management

**DOI:** 10.3389/fmed.2022.987011

**Published:** 2022-08-01

**Authors:** Naveen Kumar Devanga Ragupathi, Balaji Veeraraghavan, Esther Karunakaran, Peter N. Monk

**Affiliations:** ^1^Department of Chemical and Biological Engineering, The University of Sheffield, Sheffield, United Kingdom; ^2^Biofilms and Antimicrobial Resistance Consortium of ODA Receiving Countries (BARCOD), The University of Sheffield, Sheffield, United Kingdom; ^3^Sheffield Collaboratorium for Antimicrobial Resistance and Biofilms (SCARAB), The University of Sheffield, Sheffield, United Kingdom; ^4^Division of Microbial Interactions, Bioberrys Healthcare and Research Centre, Vellore, India; ^5^Department of Clinical Microbiology, Christian Medical College, Vellore, India; ^6^Department of Infection, Immunity and Cardiovascular Disease, The University of Sheffield, Sheffield, United Kingdom

**Keywords:** biofilm, nosocomial infection, antimicrobial resistance, disease surveillance, alternative therapies

Antimicrobial resistance (AMR) is a global health threat recently. AMR also implies a significant cost to the economy. Longer hospital stays due to prolonged illness are mainly due to persistent infections. These infections are largely due to the ability of the bacteria to thrive in patients with associated medical devices by their biofilm forming ability. Biofilms are complex matrix-like structures created by aggregating cells and secretions that increase AMR through a variety of mechanisms.

AMR occurs due to changes in the microorganisms over time leading to a lack of response to treatment (1). The phenomenon can be due to acquired resistance through plasmids or development of intrinsic resistance due to mutations in their genomes (2). There are far fewer choices of antimicrobials available to combat such multi-drug resistant organisms.

Biofilm-associated development of AMR is due to factors such as decreased drug penetration, increased drug damage due to secretions in biofilm matrix and changes in microbial metabolism. Biofilm formation also results in exchange of AMR genes in a polymicrobial environment (3). However, there is only very limited information available on biofilms and the development of AMR.

According to NIH and CDC, up to 80% of the total number of microbial infections and >60% of nosocomial infections are due to biofilms (4–6). These biofilm infections not only associate with medical devices such as catheters, ventilators, prostheses and contact lenses but also affect the mucosal layer of digestive and respiratory tracts.

## Importance of biofilms in clinical scenarios

Biofilms are significant because of their ability to exhibit resistance to antibiotics and antifungals through their complex microbial colony structure, which enhances inter- and intra-species exchange of AMR genes, ensures protection from antimicrobial penetration and enhances persistence (7). Bacteria present in biofilms were reported to exhibit 10–1,000 times antimicrobial resistance in comparison to their planktonic counterparts (8).

Currently, clinicians empirically treat biofilm-mediated infections with prolonged high doses of a combination of antibiotics which can lead to a further rise of AMR. Although several reports have been published on biofilms and antimicrobial resistance individually, there is still a lacuna of reports pertaining to significant association of these factors, and their contribution in increasing AMR burden. Given the consequences and prevalence of biofilm mediated nosocomial infections, it is important to push for the advancement and application of novel treatments.

The purpose of this Research Topic is to provide a brief background on biofilm-mediated infections in clinical scenario, and to summarize recent research work and improvements to tackle biofilms and their treatment strategies with alternatives to way forward.

A review article by Roy et al. explains the convergence of biofilm and AMR in *A. baumannii* infections. *A. baumannii* is well-known for its ability to acquire AMR determinants easily and will thrive on both biotic and abiotic surfaces. Multiple factors such as biofilm-associated protein, outer membrane protein A, chaperon-usher pilus, iron uptake mechanism, poly-β-(1, 6)-N-acetyl glucosamine, BfmS/BfmR two-component system, PER-1, and quorum sensing were attributed to the strong biofilm forming ability of *A. baumannii*.

Likewise, multi-pronged mechanisms such as EPS matrix, exoenzymes like ß-galactosidase and ß-lactamases for degradation of antibiotics, metal chelation, extracellular signaling, mutation in the antibiotic target site, and oxidation-mediated inactivation of antibiotics lead to antimicrobial tolerance (AMT) in *A. baumannii*. Even though the antibiotics are not completely damaged by these mechanisms, it reduces the antibiotic concentration to a sub-lethal level. Interestingly, eDNA released by the cells also play a role in AMT, where the negatively charged eDNA binds to positively charged antibiotics such as aminoglycosides and gathers antibiotics up to 25% of its weight.

Roy et al. address the correlation between biofilms and AMR in *A. baumannii* with reports suggesting that antibiotic-resistant *Acinetobacter spp*. form strong biofilms compared to susceptible bacteria. Interestingly, *A. baumannii* in ICUs and burn units showed high-biofilm forming abilities with co-production of AmpC and ESBLs leading to increased AMR. Genes reported to be associated with high-biofilm producing phenotype in MDR *A. baumannii* were *omp*A*, bfm*S, *bap, csuE, bla*__*PER*_−1_, and *epsA*. Such biofilm producing *A. baumannii* has a significant effect on patient recovery in case of ventilator-associated pneumonia (VAP), blood-stream infection (BSI), urinary-tract infection (UTI), patients with orthopedic implants and in ICUs.

Antimicrobial combinations like imipenem-rifampicin, colistin-rifampicin, imipenem-colistin-rifampicin, meropenem-sulbactam, and tigecycline-sulbactam have shown significant inhibition of *A. baumannii* biofilms. Colistin-levofloxacin, colistin-tigecycline-, and tigecycline-levofloxacin or these combinations with clarithromycin were also used as catheter lock solutions to prevent catheter-related *A. baumannii* infections (Roy et al.).

*S. epidermidis* is another clinical pathogen now known for its ability to cause nosocomial blood stream infections despite of their commensal nature. Most of these infections associate with implanted medical devices. Due to the lack of effective antibiofilm therapies, treatment usually requires removal of the device, causing a substantial increase in patient morbidity. Study by Oliveira et al. confirms the involvement of siderophores in protecting cell damage due to oxidative stress and demonstrated the *in vivo* relevance of a siderophore-mediated iron acquisition during *S. epidermidis* infections. This study is the first to address the underlying mechanisms of siderophore production in *S. epidermidis* and the role of siderophore-mediated iron acquisition to support its survival within the host.

An opinion by Shein et al. highlights the purpose of novel antibiotic therapies to overcome colistin resistance in *K. pneumoniae*. Increasing resistance to colistin in *K. pneumoniae* uropathogens due to chromosomal mutations and plasmid-mediated *mcr* genes result in chronic severe and recurrent UTI in clinical settings. *K. pneumoniae* is one of the most common causatives of UTI. Their ability to form biofilms in medical devices results in biofilm-mediated antibiotic tolerance. Shein et al. highlight the importance of exploring effective alternative therapies for treating UTIs caused by colistin-resistant *K. pneumoniae* biofilms.

Though several options are available such as plazomicin with its activity against both plasmid-mediated *mcr1* and chromosomal mutation of *pmr*AB or *pho*PQ or *mgr*B mechanism; meropenem-vaborbactam as carbapenem-β lactamase inhibitor; cefiderocol with enhanced outer membrane penetration system, the most effective strategy to prevent evolution of colistin resistance would be the combination therapy in addition to non-antibiotic medications or natural compounds. This also means that bacteriophage therapy, nanocarrier strategies and modification of urinary catheters are available future innovative treatment options for effective control of colistin-resistant *K. pneumoniae* UTIs.

A case report by Racenis et al. is an example of a recent advancement to approach biofilm-mediated infection, especially caused by an MDR organism. Here, a patient infected by MDR *P. aeruginosa* was treated with a strategic dual antimicrobial therapy of local bacteriophage application and IV ceftazidime-avibactam combined with surgical intervention and wound debridement was applied. The antibiotic concentration required to prevent biofilm was decreased to the MIC cut off value, thereby making the strain susceptible to ceftazidime-avibactam. This led to eradication of bacterial infection locally in biofilm-associated femur osteomyelitis.

## Tackling biofilm infections: Recent advancements

Recent advances have been made in exploring alternative strategies that affect biofilm lifestyle, inhibit biofilm formation, degrade biofilm components, and/or cause dispersal. Recently there is a growing interest in the use of ultrasound-mediated microbubbles (UMM), which are gaseous cores surrounded by stabilizing shells that can be used to deliver drugs and to mechanically disrupt biofilms (9). Several studies have reported that use of UMM in conjunction with antibiotics found to be more effective in clearing biofilms. In addition, advancements have been made in nanotechnology-based treatments for biofilms. Different type of nanoparticles has been used as antimicrobial and antibiofilm metal nanoparticles, organic nanoparticles, green nanoparticles, and their combinations. Several studies have demonstrated the successful application of nanoparticle-based drug delivery and they have also found enhanced penetration into the biofilms (10, 11).

In the line of newer approaches for biofilm treatment, bacteriophages can readily penetrate the biofilm matrix than conventional antibiotics (12). Phage therapy treats bacterial infections without detrimental effects to the host. For this reason, phage therapy is widely being reconsidered as a treatment option complementary to, and synergistic with antibiotics. Another interesting aspect of phage therapy is that the biofilm with polymicrobial communities can be treated with “phage cocktails” which consist of multiple phages proven to have *in vitro* efficacy against the target pathogen. Researchers have shown that phage treatment enhances the effectiveness of antibiotic concentration on bacterial biofilms *in vitro*. However, clinical trials would be necessary to determine their efficacy, and the feasibility of their large-scale application in healthcare would need to be considered.

## Conclusions: The way forward

There are several other strategies to prevent biofilm mediated antimicrobial resistance, including natural product-based antibiofilm agents, metabolites combined with antibiotics to eliminate persister cells, and electrochemical treatment. Moreover, biosafety and biocompatibility are major concerns and considerations when designing new antibiofilm therapeutic systems for *in vivo* application. Further exploration on the discussed novel antimicrobial agents, new drug delivery method for biofilms and new biofilm disruption principles altogether may shed some light on this difficult challenge.

## Author contributions

All authors listed have made a substantial, direct, and intellectual contribution to the work and approved it for publication.

## Funding

Authors were funded by GCRF networking grant (GCRFNGR5293) and the Academy of Medical Sciences, UK. ND is funded by a Research England QR Global Challenges Research Fellowship Award (10065043), The University of Sheffield, United Kingdom.

## Conflict of interest

The authors declare that the research was conducted in the absence of any commercial or financial relationships that could be construed as a potential conflict of interest.

## Publisher's note

All claims expressed in this article are solely those of the authors and do not necessarily represent those of their affiliated organizations, or those of the publisher, the editors and the reviewers. Any product that may be evaluated in this article, or claim that may be made by its manufacturer, is not guaranteed or endorsed by the publisher.
